# Identification of Genes Universally Differentially Expressed in Gastric Cancer

**DOI:** 10.1155/2021/7326853

**Published:** 2021-01-21

**Authors:** Yidan Shi, Lishuang Qi, Haifeng Chen, Jiahui Zhang, Qingzhou Guan, Jun He, Meifeng Li, Zheng Guo, Haidan Yan, Ping Li

**Affiliations:** ^1^Department of Bioinformatics, Key Laboratory of Ministry of Education for Gastrointestinal Cancer, School of Basic Medical Sciences, Fujian Medical University, Fuzhou 350122, China; ^2^Key Laboratory of Medical Bioinformatics, Fujian Province, Fuzhou, China; ^3^Department of Systems Biology, College of Bioinformatics Science and Technology, Harbin Medical University, Harbin 150086, China; ^4^Department of General Surgery, Fuzhou Second Hospital Affiliated to Xiamen University, 350007, China; ^5^Department of Gastric Surgery, Fujian Medical University Union Hospital, No. 29 Xinquan Road, Fuzhou 350001, China

## Abstract

Owing to the remarkable heterogeneity of gastric cancer (GC), population-level differentially expressed genes (DEGs) identified using case-control comparison cannot indicate the dysregulated frequency of each DEG in GC. In this work, first, the individual-level DEGs were identified for 1,090 GC tissues without paired normal tissues using the RankComp method. Second, we directly compared the gene expression in a cancer tissue to that in paired normal tissue to identify individual-level DEGs among 448 paired cancer-normal gastric tissues. We found 25 DEGs to be dysregulated in more than 90% of 1,090 GC tissues and also in more than 90% of 448 GC tissues with paired normal tissues. The 25 genes were defined as universal DEGs for GC. Then, we measured 24 paired cancer-normal gastric tissues by RNA-seq to validate them further. Among the universal DEGs, 4 upregulated genes (*BGN*, *E2F3*, *PLAU*, and *SPP1*) and 1 downregulated gene (*UBL3*) were found to be cancer genes already documented in the COSMIC or F-Census databases. By analyzing protein-protein interaction networks, we found 12 universally upregulated genes, and we found that their 284 direct neighbor genes were significantly enriched with cancer genes and key biological pathways related to cancer, such as the MAPK signaling pathway, cell cycle, and focal adhesion. The 13 universally downregulated genes and 16 direct neighbor genes were also significantly enriched with cancer genes and pathways related to gastric acid secretion. These universal DEGs may be of special importance to GC diagnosis and treatment targets, and they may make it easier to study the molecular mechanisms underlying GC.

## 1. Introduction

In 2012, there were 951,600 estimated new cases and 723,100 deaths from gastric cancer (GC) [1]. Although current therapies for GC, including surgery, radiation, and chemotherapy, may improve patient survival, the 5-year survival rate of GC is still less than 30% [2]. The interindividual molecular heterogeneity of GC is a large obstacle to its clinical diagnosis and treatment. Identifying common molecular biomarkers of GC is of particular significance. Currently, various methods, such as SAM [3], limma [4, 5], and RankProd [6], have been used to identify population-level differentially expressed genes (DEGs) between a group of GC tissues and a group of normal controls. However, the interindividual heterogeneity of DEGs was not considered in these methods. Therefore, for a given population-level DEG, we cannot know whether it was frequently dysregulated in any particular type of cancer.

Currently, three methods have been proposed to tackle this difficulty. First, the differential expression status of each gene was determined in a given cancer tissue by comparing the average level of expression of the same gene in a group of normal tissues [7–9]. However, because the gene expression levels of normal tissues vary profoundly across individuals [10–12], this method may easily be affected by biological variations. Second, paired cancer-normal samples were used to identify individual-level DEGs by comparing the expression level of a given gene in a cancer tissue with that in its paired normal tissue [13]. Because paired cancer-normal samples are relatively scarce, the scope of application of this method has been limited. In any case, paired cancer-normal samples are valuable and reliable specimens for individual analysis. Third, we previously developed an algorithm, named RankComp, to identify the DEGs in each cancer tissue by comparing the relative expression orderings (REOs) in cancer tissue to the highly stable REOs predetermined in a large collection of normal tissues [14]. This method allows us to identify DEGs for each cancer tissue in the absence of its paired normal tissue. To identify robust common biomarkers for GC, we not only analyzed samples with paired cancer-normal gastric tissues but also analyzed samples with only GC tissues using RankComp.

In this study, we aimed to identify genes universally dysregulated in GC, which might affect the carcinogenesis of cancer. Using large sample data collected from different sources, 25 reliable universal DEGs were identified for GC. These genes were cancer genes or closely related to cancer genes and the pathways involved in cancer. These 25 genes may be of special importance to GC diagnosis and treatment targets and can facilitate the study of the molecular mechanism of GC.

## 2. Methods

### 2.1. Samples and Data Measurement

We measured the gene expression profiles for 24 paired cancer-normal gastric tissues. All gastric tissue samples were collected from surgical resection and were frozen while fresh. TRIzol reagent (Invitrogen) was used to isolate total RNA from fresh frozen tissues according to the manufacturer's protocol. We used 1–2 *μ*g of total RNA for mRNA capture using the NEBNextPolyA mRNA Magnetic Isolation Module. Using a NEBNext Ultra Directional RNA Library Prep Kit, the stranded RNA-seq libraries were constructed. The 2 × 150 paired-end sequencing was performed on an Illumina HiSeqXten (Illumina, USA). We preprocessed the raw RNA-seq files (.fastq) by Trimmomatic [15], and we aligned reads to the reference genome (GRCh37) using hisat2 [16]. Finally, the reads per kilobase per million mapped read (RPKM) values of genes were computed and used to represent the expression levels of genes using StringTie [17]. The study was approved by the Fujian Medical University Union Hospital Institutional Review Board, and written consent forms were obtained from all participants.

### 2.2. Public Data and Preprocessing

Gene expression profiles of gastric tissues were downloaded from the Gene Expression Omnibus (GEO) database [18] and the Cancer Genome Atlas (TCGA) data portal (http://tcga-data.nci.nih.gov/tcga/) [19]. The datasets, including paired cancer-normal gastric tissues and GC tissues without paired normal tissues, were collected in this study (Table 1). We provide a flow diagram for the inclusion and exclusion criteria of this study and explain how we chose the DEGs in Supplementary Figure 1.

For the gene expression profiles measured using the Affymetrix platform, we only downloaded the raw data (.CEL files). These raw data were processed using the Robust Multi-array Average algorithm for background adjustment without quantile normalization [20]. For the datasets measured using the Illumina platform, the processed data were directly downloaded for the following analysis. Finally, we mapped each probe ID to Entrez gene ID using the corresponding platform annotation file for the array-based data (Affymetrix and Illumina platforms). If a gene was mapped by multiple probes, the expression level of this gene was calculated as the arithmetic mean of these probes. For GC tissues measured by RNA-seq, the RPKM or FPKM values of genes were downloaded for the following analysis.

### 2.3. Identification of Population-Level DEGs

The Student *t*-test was used to identify DEGs between GC tissues and GC-adjacent normal tissues. Here, we identified two lists of DEGs using two independent datasets and evaluated the concordance score of the two lists of DEGs by the cumulative binomial distribution model [21]. The DEGs reproducibly detected by the two independent datasets were here considered population-level DEGs for GC.

### 2.4. Identification of Individual-Level DEGs

Here, we only analyzed the individual-level differential expression status for population-level DEGs to confirm that the DEGs were involved in GC. First, a total of 74 normal gastric tissues measured by Affymetrix GPL570 and 32 normal gastric tissues measured by HiSeq_RNASeqV2 were used to identify significantly stable REOs for the two platforms, respectively. For normal tissues measured by a particular platform, a binomial distribution test was used to identify significantly stable REOs [22]. The consistent stable REOs in both Affymetrix GPL570 and HiSeq_RNASeqV2 were defined as crossplatform REOs. Based on the crossplatform stable REOs identified using normal gastric tissues, RankComp was used to identify DEGs in a cancer tissue compared to its own previously normal state. A detailed description of the RankComp algorithm is given in Wang et al. [14]. Paired cancer-normal gastric tissues were also used to identify individual-level DEGs for each cancer tissue. A gene was defined as upregulated if its expression level in the cancer tissue was higher than in the paired normal tissue. The gene was defined as downregulated if the reverse was true. We then calculated the frequency of upregulation and downregulation for each DEG. The 95% confidence interval (CI) of the upregulated or downregulated frequency of each DEG was also calculated by the binom.confint algorithm in R language.

### 2.5. Pathway Enrichment Analysis

The 223 pathways covering 6290 unique genes were downloaded from the KEGG database on October 21, 2018 [23]. Here, the human disease pathways were excluded from this study. The hypergeometric distribution model was used to determine biological pathways that were significantly enriched with genes of interest [24].

### 2.6. Network Analysis

Protein-protein interaction (PPI) data were collected from the SIGnaling Network Open Resource [25] database and a literature-curated human signaling network (version 6, http://www.cancer-systemsbiology.org/HuamnSignalingNet_v6.csv) [26]. A nonredundant PPI network containing 6,920 proteins and 72,840 interactions was further extracted for our analysis.

## 3. Results

### 3.1. Analysis of Population-Level DEGs

First, we used two independent datasets (GSE29272 and GSE29998) to identify the population-level DEGs. Using the paired Student's *t*-test with 5% FDR control, 9,340 and 4,928 DEGs were identified from GSE29272 and GSE29998, respectively (Supplementary Table S1). The two lists of DEGs had 3,002 overlaps, and 93.23% (2,799) of the overlapped genes showed the same dysregulated directions in the GC tissues compared with the paired normal tissues (binomial test, *P* < 1.0 × 10^−16^). Then, the 2,799 reproducible DEGs were defined as the population-level DEGs for GC.

**Figure 1:**
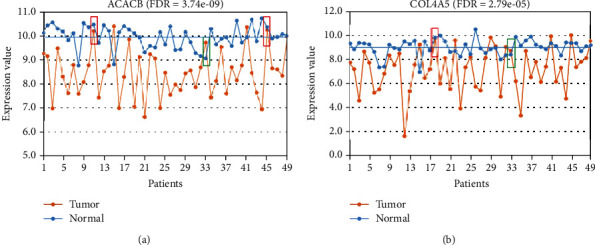
The expression levels of ACACB (a) and COL4A5 (b) in the 49 paired cancer-normal gastric tissues from GSE29998.

Owing to the heterogeneity of cancer, a given DEG detected at the population level cannot tell us whether it is dysregulated in a particular cancer tissue or not. To address this difficulty, some studies have compared the expression levels of genes in a given cancer tissue to the average expression levels of the same genes in a set of normal tissues [7–9]. However, this method may easily be affected by biological variations because of the large interindividual variations of gene expression in normal tissues [10–12]. For example, in 49 paired cancer-normal gastric tissues from GSE29998, the gene *ACACB*, which is downregulated at the population level in GC, was identified as upregulated in 4 of the 49 GC tissues relative to its average expression level in the 49 paired normal gastric tissues (Figure 1(a)). However, *ACACB* should be identified as downregulated in two of the four GC tissues when compared to the expression levels in the corresponding paired normal tissues. As shown in the green box in Figure 1(a), the level of expression of *ACACB* was higher in the GC tissue than in the paired normal tissue but below the average expression level in the normal tissues, and so it could not be identified as upregulated in the GC tissue. A similar phenomenon was also observed in another example of gene *COL4A5* (Figure 1(b)).

### 3.2. Universal Upregulated and Downregulated Genes in GC Tissues

A total of 1,090 GC tissues without paired normal tissues and 448 GC tissues with paired normal tissues were collected from the GEO and TCGA databases (Table 1). Here, we defined the DEGs that were dysregulated in at least 90% of GC tissues without paired normal tissues and also in 90% of paired cancer-normal tissues as universal DEGs for GC. Considering the existence of measurement variations and low quality of tissue samples, we defined the genes dysregulated in at least 90% of GC tissues as universal DEGs rather than reserving the term for those dysregulated in 100% of GC tissues. To ensure that the identified DEGs are involved in GC, we only analyzed the 2,799 population-level DEGs of GC.

**Figure 2:**
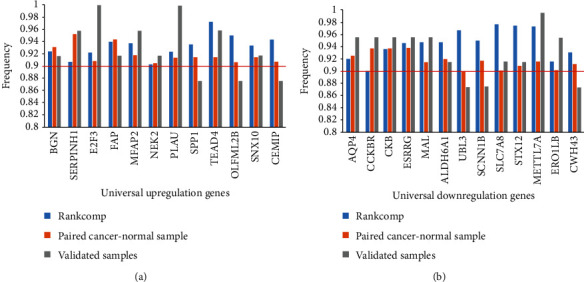
The dysregulated frequencies of the 25 universal DEGs in 1,990 GC tissues, 448 paired cancer-normal tissues, and 24 paired cancer-normal tissues measured by us, respectively. (a) The upregulated frequencies. (b) The downregulated frequencies.

Using 74 normal gastric tissues detected by Affymetrix GPL570 and 32 normal gastric tissues detected by HiSeq_RNASeqV2 (Table 1), 195,153,494 and 182,078,993 significantly stable REOs were identified for the two platforms (binomial test, FDR < 0.05), respectively. Among the 105,408,410 overlapping REOs identified in both platforms, all showed the same REO patterns (binomial test, *P* < 1.0 × 10^−16^). Based on these 105,408,410 crossplatform stable REOs, RankComp was used to identify the differential expression statuses of the 2,799 population-level DEGs in each of the 1,090 GC tissues (Methods). The 448 paired cancer-normal gastric tissues, pooled from 14 datasets measured using 10 different platforms, were used to identify individual-level DEGs by comparison of the gene expression levels between a given cancer tissue and its paired normal tissue. The frequency of upregulation and downregulation in the 448 GC tissues and the 95% CI of the frequency were calculated for each of the 2,799 population-level DEGs. Finally, 25 DEGs, including 12 upregulated and 13 downregulated genes, were observed in at least 90% of the 1,090 GC tissues and in 90% of the 448 GC tissues (Figure 2). The 25 genes were defined as universal DEGs for GC.

To further validate these universal DEGs, we measured gene expression profiles for 24 paired cancer-normal gastric tissues using RNA-seq. Compared with paired normal gastric tissues, 9 of the 12 universal upregulated genes were upregulated in more than 90% of the 24 GC tissues. The other three genes, *CEMIP*, *OLFML2B*, and *SPP1*, were also upregulated in 88% (21) of the 24 GC tissues (Figure 2). Among the 13 universal downregulated genes, 9 genes were validated in more than 90% of the 24 paired cancer-normal tissues. The remaining three genes were also validated in 88% (21) of the 24 paired cancer-normal tissues (Figure 2).

### 3.3. Functional Analysis of Universal Genes

To further analyze the biological functions of common DEGs in GC, we mapped the 25 universal DEGs to the PPI network (Methods). The 25 universal DEGs and corresponding 300 direct neighbor genes with interaction relations were extracted as a new subnetwork for the following analysis.

**Figure 3:**
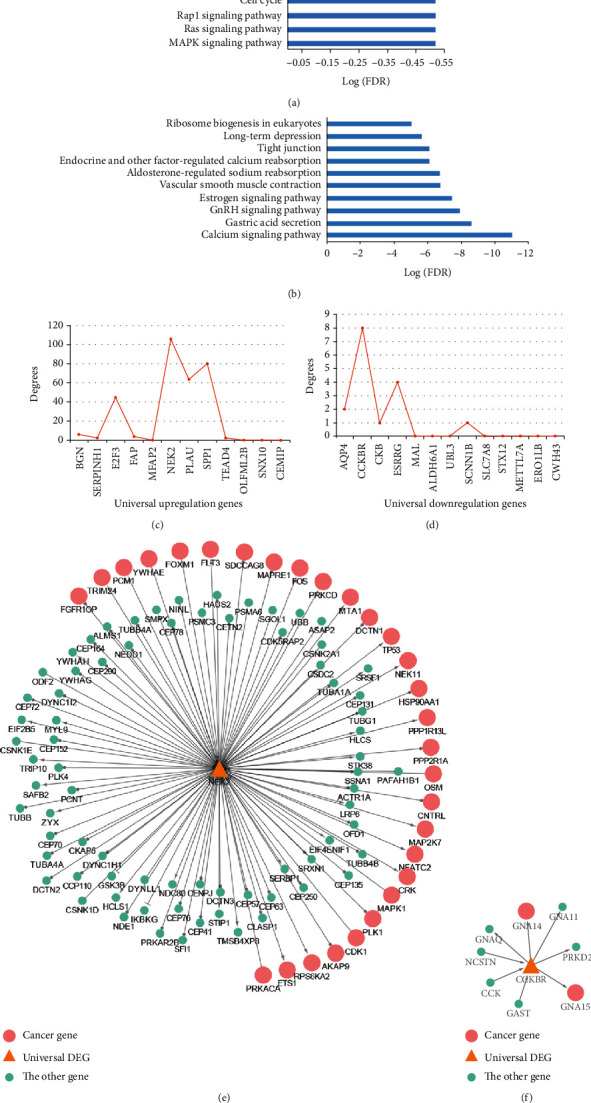
Functional analysis of upregulated and downregulated genes, respectively. The top 10 pathways enriched with upregulated (a) and downregulated (b) genes. (c) The degrees of upregulated genes. (d) The degrees of downregulated genes. (e) The subnetwork contains NEK2 and its direct neighbor genes. (f) The subnetwork contains CCKBR and its direct neighbor genes.

For the 12 upregulated genes, 284 genes were directly linked to them. The 296 genes were significantly enriched in 56 pathways (Supplementary Table S2), such as MAPK signaling pathway, cell cycle, and focal adhesion (Figure 3(a), hypergeometric distribution model, FDR < 0.05). Moreover, the 296 genes were significantly enriched with cancer genes documented in COSMIC [27] or F-Census database [28] (hypergeometric distribution model, *P* < 0.05). Here, 4 of the 12 upregulated genes, *BGN*, *E2F3*, *PLAU*, and *SPP1*, were cancer genes. Among the top 4 genes (*NEK2*, *SPP1*, *PLAU*, *E2F3*) with the largest degrees (Figure 3(c)), 3 were cancer genes; the gene *NEK2* is not. *NEK2* had 106 direct neighbor genes, among which 30 were cancer genes (Figure 3(e)). Moreover, *NEK2* was found to be frequently upregulated across multiple cancers [13]. *NEK2*, which is a serine/threonine-protein kinase, is involved in mitotic regulation [29]. The overexpression of *NEK2* can increase the ability of *Mad2* to cause delays in cell division [30] The dysregulation of *NEK2* may play important roles in tumorigenesis and may be an effective target for cancer treatment [31–33].

Another 16 genes were directly linked to the 13 downregulated genes. The 29 genes were significantly enriched in 15 pathways (Supplementary Table S2), such as calcium signaling pathway and gastric acid secretion (Figure 3(b), hypergeometric distribution model, FDR < 0.05). Eight of the 29 genes were cancer genes documented in COSMIC [27] or F-Census [28], which cannot happen by chance (hypergeometric distribution model, *P* < 0.05). As shown in Figure 3, *CCKBR* was the gene with the largest degree among the universally downregulated genes and it interacted directly with two cancer genes (Figures 3(d), 3(f)). *CCKBR* has been reported to play important roles in gastric carcinogenesis by regulating stem cell function and epithelial homeostasis [34].

Hypermethylation of CpG sites within the promoter of a gene is an important event leading to its downregulation [35]. Here, we collected DNA methylation profiles of 115 paired cancer-normal gastric tissues from GSE30601 and GSE25869 datasets. A gene was defined as hypermethylated when more than 50% of the 115 GC tissues had a higher methylation level than in the paired normal tissues. Among the 13 downregulated genes, 12 genes with DNA methylation information were analyzed. We found that 10 universal downregulated genes were hypermethylated in 49% of the 115 GC tissues, and 2 genes, *ESRRG* and *AQP4*, showed higher methylation levels in 41% of the 115 GC tissues (Supplementary Table S3). The results showed that hypermethylation of gene promoters may drive events for the downregulation of the 13 universally downregulated genes.

## 4. Discussion

In this study, we aimed to find genes universally dysregulated in all or almost all GC patients to deal with interindividual heterogeneity. To make full use of all gastric cancer data, we identified individual-level DEGs for GC tissues with and without paired normal tissues. To find reliable common biomarkers for GC, we only used DEGs found in 90% of paired cancer-normal gastric tissues and also in 90% of GC tissues without paired normal tissues. Owing to the strict control, we may have missed some important universally dysregulated genes. Among the 25 universal DEGs, only the 12 universally downregulated genes with DNA methylation information could be explained by changes in DNA methylation. Among the 12 universal upregulated genes, possible causal events should be further investigated using large-sample multidimensional data to deepen our understanding of the mechanism underlying carcinogenesis.

Notably, only 1 gene (*NEK2*) of the 25 universal DEGs was found to be upregulated in at least 90% of 649 cancer tissues in pan-cancer analysis across 23 cancer types [13]. Moreover, Li et al. showed that *NEK2* was highly expressed in GC cell lines and related to promoting cell proliferation, migration, and tumor growth [36]. As shown in Table S4 and S5, the remaining universal DEGs may be cancer markers or involved in immunity, cell proliferation, gastrin, and cholecystokinin. For example, *FAP* has been thought to be involved in the control of fibroblast growth or epithelial-mesenchymal interactions during development, tissue repair, and epithelial carcinogenesis. *BGN* may regulate inflammation and innate immunity. *SNX10* may play a role in regulating endosome homeostasis, and Ye et al. found that its upregulation may lead to gastric acidification defects [37]. *SLC7A8*, also known as *LAT2*, is a member of the L-type amino acid transporter (*LAT*) family. Although the *LAT* family has been reported to play important roles in cancer development [38–41], few studies have validated the role of *LAT2* in cancer cell growth. The biological functions of some universal DEGs should be studied further in the future, which may help establish mechanisms underlying GC.

## 5. Conclusion

In summary, these common DEGs may be of particular importance to the development and progression of GC, which may be key diagnostic and treatment targets.

## Figures and Tables

**Table 1:** The public datasets used in this study.

Datasets	Platforms	The number of normal samples	The number of cancer samples
Datasets with paired cancer-normal samples			
GSE29272	Affymetrix GPL96	134	134
GSE2685	Affymetrix GPL80	6	6
GSE63089	Affymetrix GPL5175	45	45
GSE13195	Affymetrix GPL5175	25	25
GSE56807	Affymetrix GPL5175	5	5
GSE13911	Affymetrix GPL570	31	31
GSE19826	Affymetrix GPL570	12	12
GSE79973	Affymetrix GPL570	10	10
GSE65801	Agilent GPL14550	32	32
GSE51575	Agilent GPL13607	26	26
GSE29998	Illumina GPL6947	49	49
GSE13861	Illumina GPL6884	19	19
GSE63288	SOLiD GPL13393	22	22
TCGA	HiSeq_RNASeqV2	32	32
Datasets without paired cancer-normal samples			
GSE54129	Affymetrix GPL570	21	111
GSE38749	Affymetrix GPL570	-	15
GSE57303	Affymetrix GPL570	-	70
GSE34942	Affymetrix GPL570	-	56
GSE22377	Affymetrix GPL570	-	43
GSE35809	Affymetrix GPL570	-	70
GSE51105	Affymetrix GPL570	-	94
GSE15459	Affymetrix GPL570	-	200
GSE34942	Affymetrix GPL570	-	56
TCGA	HiSeq_RNASeqV2	-	375

Notes: “-” represented that there is no sample in the corresponding category.

## Data Availability

The datasets generated and analyzed during the current study are available from the corresponding author on reasonable request.

## Abbreviations

GC: Gastric cancer
DEGs: Differentially expressed genes
REOs: Relative expression orderings
GEO: Gene Expression Omnibus
TCGA: The Cancer Genome Atlas
RPKM: The reads per kilobase per million mapped reads
CI: Confidence interval
PPI: Protein-protein interaction.

## Supplementary Materials

Supplementary Materials Table S1: The population-level differentially expressed genes in GSE29272 and GSE29998. Table S2: The pathways enriched with universal downregulated (or upregulated) genes and their direct neighbor genes. Table S3: the proportion of samples with hypermethylation CpG sites in each of universal downregulated genes. Table S4: The summary of universal upregulated DEGs annotated from the NCBI gene database. Table S5: The summary of universal downregulated DEGs annotated from the NCBI gene database. Figure S1: The flow chart of this study.
