# Supplementary material for: Identification of Genes Universally Differentially Expressed in Gastric Cancer
Source: Biomed Res Int. 2021 Jan 21;2021:7326853. doi: 10.1155/2021/7326853 (PMC7843176; doi:10.1155/2021/7326853)
Supplement: Supplementary Materials — Table S1: The population-level differentially expressed genes in GSE29272 and GSE29998. Table S2: The pathways enriched with universal downregulated (or upregulated) genes and their direct neighbor genes. Table S3: the proportion of samples with hypermethylation CpG sites in each of universal downregulated genes. Table S4: The summary of universal upregulated DEGs annotated from the NCBI gene database. Table S5: The summary of universal downregulated DEGs annotated from the NCBI gene database. Figure S1: The flow chart of this study. [file 7326853.f1.zip › Table S1.docx]

**Supplementary Table S1.** The pupolation-level differentially expressed genes in GSE29272 and GSE29998.

| Datasets | Up-regulated genes | Down-regulated genes | Overlaps-up | Overlaps-down | Concordance score |
| --- | --- | --- | --- | --- | --- |
| GSE29272 | 4267 | 5073 | 1702 | 1097 | 93.23% |
| GSE29998 | 2981 | 1947 |  |  |  |

Overlaps-up and Overlaps-down represent the numbers of overlapped up-regulated and down-regulated genes, respectively.
