# Supplementary material for: Identification of Genes Universally Differentially Expressed in Gastric Cancer
Source: Biomed Res Int. 2021 Jan 21;2021:7326853. doi: 10.1155/2021/7326853 (PMC7843176; doi:10.1155/2021/7326853)
Supplement: Supplementary Materials — Table S1: The population-level differentially expressed genes in GSE29272 and GSE29998. Table S2: The pathways enriched with universal downregulated (or upregulated) genes and their direct neighbor genes. Table S3: the proportion of samples with hypermethylation CpG sites in each of universal downregulated genes. Table S4: The summary of universal upregulated DEGs annotated from the NCBI gene database. Table S5: The summary of universal downregulated DEGs annotated from the NCBI gene database. Figure S1: The flow chart of this study. [file 7326853.f1.zip › Table S2.docx]

**Table S2.** The pathways enriched with universal up- and down-regulation genes and their direct neighbor genes.

| universal up-regulation genes and their direct neighbor genes | |
| --- | --- |
| Pathway | FDR |
| MAPK signaling pathway | 0 |
| ErbB signaling pathway | 2.07e-05 |
| Ras signaling pathway | 0 |
| Rap1 signaling pathway | 0 |
| cGMP-PKG signaling pathway | 0.044 |
| cAMP signaling pathway | 0.029 |
| Chemokine signaling pathway | 0.003 |
| NF-kappa B signaling pathway | 4.56e-04 |
| HIF-1 signaling pathway | 4.45e-04 |
| FoxO signaling pathway | 1.80e-06 |
| Sphingolipid signaling pathway | 0.012 |
| Cell cycle | 0 |
| Oocyte meiosis | 2.86e-06 |
| p53 signaling pathway | 1.87e-08 |
| Endocytosis | 0.010 |
| Phagosome | 1.25e-06 |
| mTOR signaling pathway | 0.002 |
| PI3K-Akt signaling pathway | 0 |
| AMPK signaling pathway | 0.016 |
| Apoptosis | 1.60e-05 |
| Wnt signaling pathway | 1.17e-05 |
| Dorso-ventral axis formation | 0.016 |
| TGF-beta signaling pathway | 4.47e-06 |
| VEGF signaling pathway | 5.44e-05 |
| Osteoclast differentiation | 1.34e-12 |
| Hippo signaling pathway | 7.89e-06 |
| Focal adhesion | 0 |
| ECM-receptor interaction | 2.37e-13 |
| Cell adhesion molecules (CAMs) | 0.014 |
| Adherens junction | 1.20e-05 |
| Gap junction | 1.60e-05 |
| Signaling pathways regulating pluripotency of stem cells | 4.18e-05 |
| Complement and coagulation cascades | 0.013 |
| Platelet activation | 2.75e-04 |
| Toll-like receptor signaling pathway | 2.44e-09 |
| NOD-like receptor signaling pathway | 0.005 |
| Jak-STAT signaling pathway | 0.002 |
| Hematopoietic cell lineage | 7.14e-08 |
| Natural killer cell mediated cytotoxicity | 7.97e-04 |
| T cell receptor signaling pathway | 5.04e-08 |
| B cell receptor signaling pathway | 1.39e-05 |
| Fc epsilon RI signaling pathway | 5.44e-06 |
| Fc gamma R-mediated phagocytosis | 4.56e-04 |
| TNF signaling pathway | 5.50e-11 |
| Leukocyte transendothelial migration | 0.001 |
| Neurotrophin signaling pathway | 6.15e-08 |
| Regulation of actin cytoskeleton | 0 |
| Insulin signaling pathway | 0.015 |
| GnRH signaling pathway | 0.001 |
| Progesterone-mediated oocyte maturation | 3.50e-06 |
| Estrogen signaling pathway | 1.16e-05 |
| Prolactin signaling pathway | 3.12e-04 |
| Thyroid hormone signaling pathway | 7.36e-06 |
| Oxytocin signaling pathway | 1.27e-05 |
| Aldosterone-regulated sodium reabsorption | 0.014 |
| Vasopressin-regulated water reabsorption | 0.001 |
| universal down-regulation genes and their direct neighbor genes | |
| Ribosome biogenesis in eukaryotes | 0.030 |
| Calcium signaling pathway | 4.72e-04 |
| NF-kappa B signaling pathway | 0.046 |
| Vascular smooth muscle contraction | 0.009 |
| Adherens junction | 0.031 |
| Tight junction | 0.015 |
| Gap junction | 0.043 |
| Long-term depression | 0.020 |
| Inflammatory mediator regulation of TRP channels | 0.049 |
| Insulin secretion | 0.042 |
| GnRH signaling pathway | 0.004 |
| Estrogen signaling pathway | 0.006 |
| Aldosterone-regulated sodium reabsorption | 0.009 |
| Endocrine and other factor-regulated calcium reabsorption | 0.014 |
| Gastric acid secretion | 0.003 |
