# Supplementary material for: Identification of Genes Universally Differentially Expressed in Gastric Cancer
Source: Biomed Res Int. 2021 Jan 21;2021:7326853. doi: 10.1155/2021/7326853 (PMC7843176; doi:10.1155/2021/7326853)
Supplement: Supplementary Materials — Table S1: The population-level differentially expressed genes in GSE29272 and GSE29998. Table S2: The pathways enriched with universal downregulated (or upregulated) genes and their direct neighbor genes. Table S3: the proportion of samples with hypermethylation CpG sites in each of universal downregulated genes. Table S4: The summary of universal upregulated DEGs annotated from the NCBI gene database. Table S5: The summary of universal downregulated DEGs annotated from the NCBI gene database. Figure S1: The flow chart of this study. [file 7326853.f1.zip › Table S3.docx]

**Supplementary Table S3.** the proportion of samples with hypermethylation CpG sites in each of universal down-regulation genes.

| Gene symbol | Proportions |
| --- | --- |
| CWH43 | 72.17% |
| METTL7A | 69.57% |
| SCNN1B | 68.70% |
| CKB | 61.74% |
| MAL | 60.00% |
| CCKBR | 59.13% |
| SLC7A8 | 58.26% |
| ALDH6A1 | 56.52% |
| STX12 | 55.65% |
| UBL3 | 51.30% |
| ESRRG | 48.70% |
| AQP4 | 40.87% |
| ERO1LB | - |
