# Supplementary material for: Identification of Genes Universally Differentially Expressed in Gastric Cancer
Source: Biomed Res Int. 2021 Jan 21;2021:7326853. doi: 10.1155/2021/7326853 (PMC7843176; doi:10.1155/2021/7326853)
Supplement: Supplementary Materials — Table S1: The population-level differentially expressed genes in GSE29272 and GSE29998. Table S2: The pathways enriched with universal downregulated (or upregulated) genes and their direct neighbor genes. Table S3: the proportion of samples with hypermethylation CpG sites in each of universal downregulated genes. Table S4: The summary of universal upregulated DEGs annotated from the NCBI gene database. Table S5: The summary of universal downregulated DEGs annotated from the NCBI gene database. Figure S1: The flow chart of this study. [file 7326853.f1.zip › Table S4.docx]

**Table S4.** The summary of universal up-regulated DEGs annotated from the NCBI gene database.

| Gene name | Summary |
| --- | --- |
| BGN | The preproprotein encoded by this gene is proteolytically processed to generate the mature protein, which plays a role in bone growth, muscle development and regeneration, and collagen fibril assembly in multiple tissues. This protein may also regulate inflammation and innate immunity. Additionally, the encoded protein may contribute to atherosclerosis and aortic valve stenosis in human patients. |
| SERPINH1 | The protein encoded by this gene is localized to the endoplasmic reticulum and plays a role in collagen biosynthesis as a collagen-specific molecular chaperone. Autoantibodies to the encoded protein have been found in patients with rheumatoid arthritis. Expression of this gene may be a marker for cancer, and nucleotide polymorphisms in this gene may be associated with preterm birth caused by preterm premature rupture of membranes. |
| E2F3 | The encoded protein by this gene recognizes a specific sequence motif in DNA and interacts directly with the retinoblastoma protein (pRB) to regulate the expression of genes involved in the cell cycle. Altered copy number and activity of this gene have been observed in a number of human cancers. |
| FAP | It is selectively expressed in reactive stromal fibroblasts of epithelial cancers, granulation tissue of healing wounds, and malignant cells of bone and soft tissue sarcomas. This protein is thought to be involved in the control of fibroblast growth or epithelial-mesenchymal interactions during development, tissue repair, and epithelial carcinogenesis. |
| MFAP2 | Microfibrillar-associated protein 2 is a major antigen of elastin-associated microfibrils and a candidate for involvement in the etiology of inherited connective tissue diseases. |
| NEK2 | This gene encodes a serine/threonine-protein kinase that is involved in mitotic regulation. This protein is localized to the centrosome, and undetectable during G1 phase, but accumulates progressively throughout the S phase, reaching maximal levels in late G2 phase. |
| PLAU | The encoded preproprotein is proteolytically processed to generate A and B polypeptide chains. These chains associate via a single disulfide bond to form the catalytically inactive high molecular weight urokinase-type plasminogen activator (HMW-uPA). HMW-uPA can be further processed into the catalytically active low molecular weight urokinase-type plasminogen activator (LMW-uPA). This low molecular weight form does not bind to the urokinase-type plasminogen activator receptor. Mutations in this gene may be associated with Quebec platelet disorder and late-onset Alzheimer's disease. |
| SPP1 | The secreted protein encoded by this gene is and binds hydroxyapatite with high affinity. The osteoclast vitronectin receptor is found in the cell membrane and may be involved in the binding to this protein. This protein is also a cytokine that upregulates expression of interferon-gamma and interleukin-12. |
| TEAD4 | This gene product is a member of the transcriptional enhancer factor (TEF) family of transcription factors, which contain the TEA/ATTS DNA-binding domain. It is preferentially expressed in the skeletal muscle, and binds to the M-CAT regulatory element found in promoters of muscle-specific genes to direct their gene expression. |
| OLFML2B | This gene encodes an olfactomedin domain-containing protein. Most olfactomedin domain-containing proteins are secreted glycoproteins. |
| SNX10 | The member of sorting nexin family encoded by this gene contain a phox (PX) domain, which is a phosphoinositide binding domain, and are involved in intracellular trafficking. This protein does not contain a coiled coil region, like some family members. This gene may play a role in regulating endosome homeostasis. |
| CEMIP | NA |
