# Supplementary material for: Identification of Genes Universally Differentially Expressed in Gastric Cancer
Source: Biomed Res Int. 2021 Jan 21;2021:7326853. doi: 10.1155/2021/7326853 (PMC7843176; doi:10.1155/2021/7326853)
Supplement: Supplementary Materials — Table S1: The population-level differentially expressed genes in GSE29272 and GSE29998. Table S2: The pathways enriched with universal downregulated (or upregulated) genes and their direct neighbor genes. Table S3: the proportion of samples with hypermethylation CpG sites in each of universal downregulated genes. Table S4: The summary of universal upregulated DEGs annotated from the NCBI gene database. Table S5: The summary of universal downregulated DEGs annotated from the NCBI gene database. Figure S1: The flow chart of this study. [file 7326853.f1.zip › Table S5.docx]

**Table S5.** The summary of universal down-regulated DEGs annotated from the NCBI gene database.

| Gene name | Summary |
| --- | --- |
| AQP4 | The protein encoded by this gene is the predominant aquaporin found in brain and has an important role in brain water homeostasis. |
| CCKBR | This gene encodes a G-protein coupled receptor for gastrin and cholecystokinin (CCK), regulatory peptides of the brain and gastrointestinal tract. This protein is a type B gastrin receptor, which has a high affinity for both sulfated and nonsulfated CCK analogs and is found principally in the central nervous system and the gastrointestinal tract. Alternative splicing results in multiple transcript variants. A misspliced transcript variant including an intron has been observed in cells from colorectal and pancreatic tumors. |
| CKB | The protein encoded by this gene reversibly catalyzes the transfer of phosphate between ATP and various phosphogens such as creatine phosphate. It acts as a homodimer in brain as well as in other tissues, and as a heterodimer with a similar muscle isozyme in heart. |
| ESRRG | It has been reported that the family member encoded by this gene functions as a transcriptional activator of DNA cytosine-5-methyltransferases 1 (Dnmt1) expression by direct binding to its response elements in the DNMT1 promoters, modulates cell proliferation and estrogen signaling in breast cancer, and negatively regulates bone morphogenetic protein 2-induced osteoblast differentiation and bone formation. |
| MAL | The protein encoded by this gene has been localized to the endoplasmic reticulum of T-cells and is a candidate linker protein in T-cell signal transduction. In addition, this proteolipid is localized in compact myelin of cells in the nervous system and has been implicated in myelin biogenesis and/or function. The protein plays a role in the formation, stabilization and maintenance of glycosphingolipid-enriched membrane microdomains. Down-regulation of this gene has been associated with a variety of human epithelial malignancies. |
| ALDH6A1 | The protein encoded by this gene is a mitochondrial methylmalonate semialdehyde dehydrogenase that plays a role in the valine and pyrimidine catabolic pathways. This protein catalyzes the irreversible oxidative decarboxylation of malonate and methylmalonate semialdehydes to acetyl- and propionyl-CoA. Methylmalonate semialdehyde dehydrogenase deficiency is characterized by elevated beta-alanine, 3-hydroxypropionic acid, and both isomers of 3-amino and 3-hydroxyisobutyric acids in urine organic acids. |
| UBL3 | NA |
| SCNN1B | Nonvoltage-gated, amiloride-sensitive, sodium channels control fluid and electrolyte transport across epithelia in many organs. These channels are heteromeric complexes consisting of 3 subunits: alpha, beta, and gamma. This gene encodes the beta subunit, and mutations in this gene have been associated with pseudohypoaldosteronism type 1 (PHA1), and Liddle syndrome. |
| SLC7A8 | NA |
| STX12 | NA |
| METTL7A | NA |
| ERO1LB | NA |
| CWH43 | NA |
