# Supplementary figures and images for: Identification of Genes Universally Differentially Expressed in Gastric Cancer
Source: Biomed Res Int. 2021 Jan 21;2021:7326853. doi: 10.1155/2021/7326853 (PMC7843176; doi:10.1155/2021/7326853)

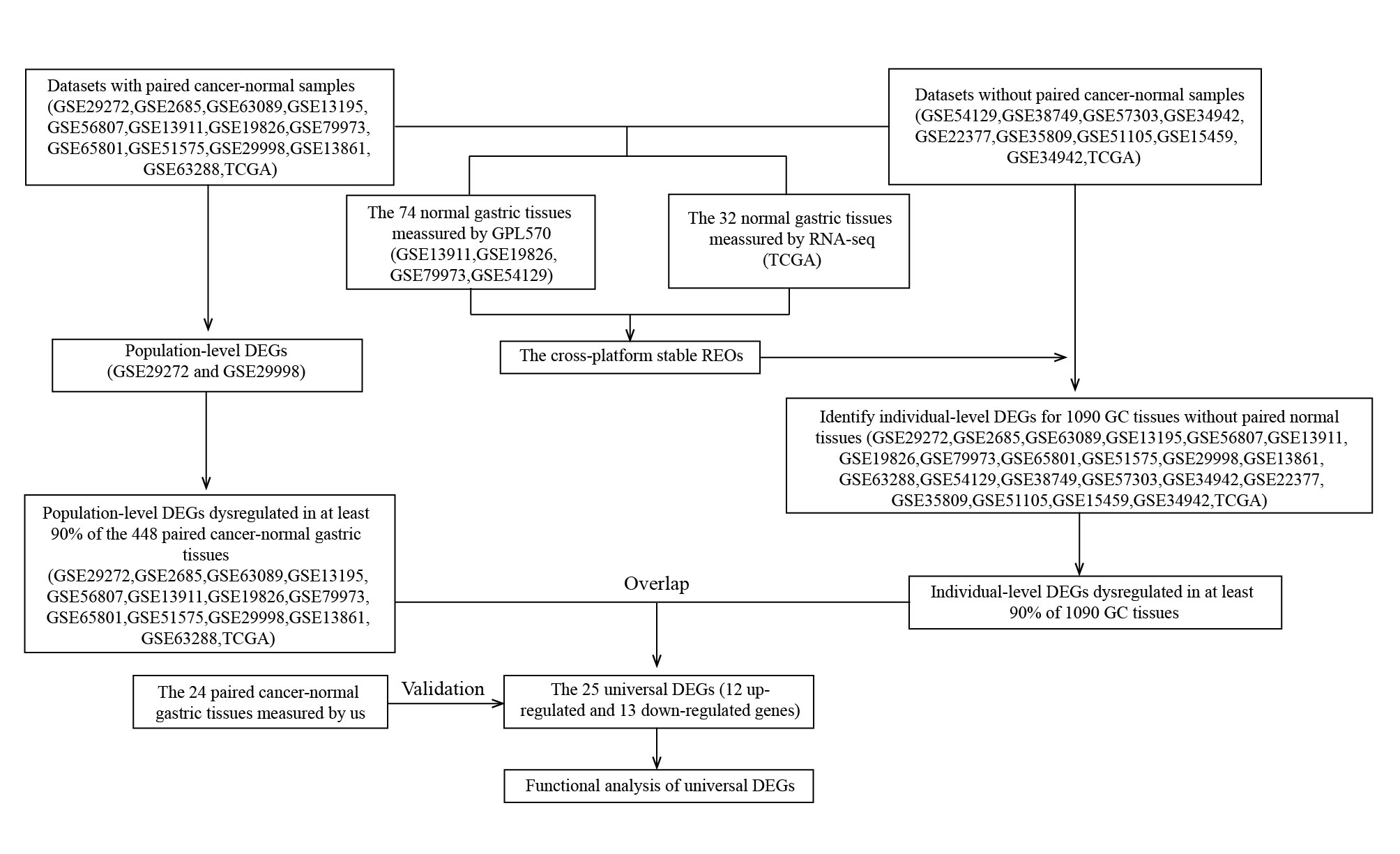

Supplement: Supplementary Materials — Table S1: The population-level differentially expressed genes in GSE29272 and GSE29998. Table S2: The pathways enriched with universal downregulated (or upregulated) genes and their direct neighbor genes. Table S3: the proportion of samples with hypermethylation CpG sites in each of universal downregulated genes. Table S4: The summary of universal upregulated DEGs annotated from the NCBI gene database. Table S5: The summary of universal downregulated DEGs annotated from the NCBI gene database. Figure S1: The flow chart of this study. [file 7326853.f1.zip › Figure_S1-01.jpg]
